# Utilization of clinical and radiological parameters to predict cognitive prognosis in patients with mild-to-moderate traumatic brain injury

**DOI:** 10.3389/fnins.2023.1222541

**Published:** 2023-07-27

**Authors:** Xi Wang, Xiaobo Hui, Xiangyu Wang, Baosheng Huang, Xiaokui Gan, Xingdong Liu, Zhiyan Shen, Yi Sun, Lixin Li

**Affiliations:** ^1^Department of Neurosurgery, The First Affiliated Hospital of Nanjing Medical University, Nanjing, China; ^2^Department of Neurosurgery, The Affiliated Huaian No. 1 People's Hospital of Nanjing Medical University, Huaian, Jiangsu, China; ^3^Department of Rehabilitation Medicine, The Affiliated Lianyungang Oriental Hospital of Kangda College of Nanjing Medical University, Lianyungang, Jiangsu, China; ^4^Department of Neurosurgery, Sir Run Run Hospital, Nanjing Medical University, Nanjing, China

**Keywords:** traumatic brain injury, cognitive impairment, predictive factor, nomogram, radiological parameters

## Abstract

**Background:**

Cognitive impairment is a common sequela following traumatic brain injury (TBI). This study aimed to identify risk factors for cognitive impairment after 3 and 12 months of TBI and to create nomograms to predict them.

**Methods:**

A total of 305 mild-to-moderate TBI patients admitted to the First Affiliated Hospital with Nanjing Medical University from January 2018 to January 2022 were retrospectively recruited. Risk factors for cognitive impairment after 3 and 12 months of TBI were identified by univariable and multivariable logistic regression analyses. Based on these factors, we created two nomograms to predict cognitive impairment after 3 and 12 months of TBI, the discrimination and calibration of which were validated by plotting the receiver operating characteristic (ROC) curve and calibration curve, respectively.

**Results:**

Cognitive impairment was detected in 125/305 and 52/305 mild-to-moderate TBI patients after 3 and 12 months of injury, respectively. Age, the Glasgow Coma Scale (GCS) score, >12 years of education, hyperlipidemia, temporal lobe contusion, traumatic subarachnoid hemorrhage (tSAH), very early rehabilitation (VER), and intensive care unit (ICU) admission were independent risk factors for cognitive impairment after 3 months of mild-to-moderate TBI. Meanwhile, age, GCS score, diabetes mellitus, tSAH, and surgical treatment were independent risk factors for cognitive impairment after 12 months of mild-to-moderate TBI. Two nomograms were created based on the risk factors identified using logistic regression analyses. The areas under the curve (AUCs) of the two nomograms to predict cognitive impairment after 3 and 12 months of mild-to-moderate TBI were 0.852 (95% CI [0.810, 0.895]) and 0.817 (95% CI [0.762, 0.873]), respectively.

**Conclusion:**

Two nomograms are created to predict cognitive impairment after 3 and 12 months of TBI. Age, GCS score, >12 years of education, hyperlipidemia, temporal lobe contusion, tSAH, VER, and ICU admission are independent risk factors for cognitive impairment after 3 months of TBI; meanwhile, age, the GCS scores, diabetes mellitus, tSAH, and surgical treatment are independent risk factors of cognitive impairment after 12 months of TBI. Two nomograms, based on both groups of factors, respectively, show strong discriminative abilities.

## Introduction

New cases of traumatic brain injury (TBI) are estimated to be 20 million per year, posing a heavy global burden of disease (Injury and Spinal Cord Injury, 2019). Based on the Glasgow Coma Scale (GCS) scores, TBI is classified as mild-to-moderate (GCS, 9–15 points) and severe (GCS,≤8 points). The incidence of mild-to-moderate TBI is much higher than that of severe TBI. Despite its low mortality, mild-to-moderate TBI still causes multiple neurological deficits in survivors (Pavlovic et al., 2019).

Cognitive impairment, a common type of neurological dysfunction after mild-to-moderate TBI, markedly worsens the quality of life and the long-term neurological outcomes in survivors (McHugh et al., 2006). Cognitive impairment after mild-to-moderate TBI is usually manifested as the impairment of executive function, memory, attention, speech, and naming (Miotto et al., 2010; Panwar et al., 2019). Cognitive function can be recovered in some TBI patients within 1–3 months post-TBI, but cognitive impairment may stay for a long term in some cases (McHugh et al., 2006). A previous study has reported that 19.2% of mild TBI patients and 39.3% of moderate TBI patients still experience cognitive impairment after 3 months of injury (Othman et al., 2022). Draper and Ponsford (2008) have revealed that cognitive impairment may persist in some patients even after 10 years of TBI, the degree of which is positively correlated with the severity of the injury.

Post-TBI cognitive impairment has been well-studied. At present, therapeutic strategies mainly include medications, rehabilitation exercises, and transcranial magnetic stimulation (TMS) (Neville et al., 2015; Jenkins et al., 2019; Martinez-Molina et al., 2022). However, efficient predictive factors for identifying high-risk populations are scant, so intervention strategies are not started promptly. In the present retrospective study, we analyzed risk factors for cognitive impairment after 3 and 12 months of mild-to-moderate TBI. Based on these factors, two nomograms were created to predict cognitive impairment in mild-to-moderate TBI patients after 3 and 12 months of injury.

## Methods

### Subjects

Mild-to-moderate TBI patients admitted to the Neurosurgery Department, the First Affiliated Hospital with Nanjing Medical University from January 2018 to January 2022 were retrospectively recruited. The inclusion criteria were as follows: (*i*) aged over 16 years old and under 90 years old; (*ii*) an interval time <24 h from TBI to admission; (*iii*) the lowest score of GCS at admission, 3 h after admission, and 6 h after admission was ≥9; and (*iv*) computed tomography (CT) of the head performed within 6 h of admission and 48 h before discharge. The exclusion criteria were as follows: (*i*) death during hospitalization; (*ii*) severe compound injuries; (*iii*) history of mental illnesses or cognitive impairments; (*iv*) hormonal disorders during the course of disease; and (*v*) status epilepticus during the course of the disease. Written informed consent was obtained from all subjects or their guardians. This study was approved by the Ethics Committee of the First Affiliated Hospital of Nanjing Medical University (No. 2022-SR-354).

### Data collection

The following data were recorded: (*i*) baseline characteristics, including sex, age, and years of education; (*ii*) medical history, including the history of hypertension, diabetes mellitus, and hyperlipidemia; (*iii*) clinical characteristics of TBI, including the GCS score and causes of injury; (*iv*) imaging features of TBI, including injury site (right, left, and both); contusions in the temporal, frontal, parietal, and occipital lobes; epidural, subdural, or traumatic subarachnoid hemorrhage (tSAH); and subdural effusion on discharge; and (*v*) treatment, including surgical treatment, ICU admission, and very early rehabilitation (VER). VER was defined as rehabilitation exercises that commenced within 3 days after admission or immediately after postoperative vital signs were stable. The GCS score was selected and recorded as the lowest score of GCS at admission, 3 h after admission, and 6 h after admission. The computed tomography (CT) examination of the TBI patient showed the presence of high-density shadow in the subarachnoid space, which was defined as tSAH. Subdural effusion was defined as effusion that appeared within 10 days of TBI with a similar uniform low-density area, width >3 mm, and CT value <20 Hu. ICU admission was defined as treatment in the intensive care unit during hospitalization regardless of the length of stay in the ICU.

### Assessment of cognitive impairment

The survivors of mild-to-moderate TBI patients were followed up for 12 months after the injury. A widely used tool to detect cognitive impairment with high sensitivity and specificity, the Montreal Cognitive Assessment (MoCA), was performed at 3 and 12 months post-TBI. The MoCA score increased by 1 point when traumatic brain injury patients had <12 years of education. Ranging from 0 to 30 points, a lower MoCA score indicated a worse cognitive function (Nasreddine et al., 2005). In the present study, cognitive impairment was defined as those with a maximal MoCA score of 26 points.

### Statistical analysis

Categorical variables were expressed as number of cases (*n*) and percentage (%). Continuous variables in a normal distribution were expressed as mean ± standard deviation ($\overset{\bar{}}{x\;}\pm$ SD); otherwise, they were expressed as median (M) and interquartile boundary values (P25, P75). Variables with a *p*-value of <0.10 in the univariable logistic regression model were introduced into the multivariable logistic regression model using the backward elimination method. The odds ratio (OR) and the corresponding 95% confidence interval (95% CI) and *p*-value were calculated. Nomograms were created using an R package by incorporating variables with a *p*-value of <0.05 identified using the multivariable logistic regression analysis. The discriminative ability of the nomogram was assessed by plotting the receiver operating characteristic (ROC) curve and calculating the C-statistics, which was equal to the area under the curve (AUC). The C-statistics ranged from 0.5 to 1.0, and a higher C-statistics indicated better discrimination of the nomogram. Internal validation of the nomogram was performed within 1,000 bootstrap resampling. A linear calibration curve indicated an acceptable goodness-of-fit of the nomogram. Statistical analysis was performed using SPSS 23.0 and packages of rms, readr, pROC, formula, and ggplot2 in R, and figures were programmatically created using R 3.6.1. A *p*-value of <0.05 was considered statistically significant.

## Results

### Clinical characteristics of subjects

A total of 365 patients with mild-to-moderate TBI were recruited. After excluding 12 deaths and 48 subjects who were lost to follow-up, 305 eligible patients were finally included in this study, including 190 with mild TBI (GCS 13–15 points) and 115 with moderate TBI (GCS 9–12 points).

After 3 months of TBI, 125 (41.0%) patients developed cognitive impairment. The incidences of cognitive impairment in mild and moderate TBI patients after 3 months of injury were 28.9% and 60.9%, respectively. Later, 52 (17.0%) TBI patients still suffered from cognitive impairment after 12 months of injury, with incidences of cognitive impairment in mild and moderate TBI patients of 8.4% and 31.3%, respectively.

### Independent risk factors for cognitive impairment after 3 and 12 months of mild-to-moderate TBI

The univariable logistic regression analysis revealed that age (*P* < 0.001), GCS scores (*P* < 0.001), >12 years of education (*P* < 0.001), hyperlipidemia (*P* < 0.001), injury side (*P* = 0.005), temporal lobe contusion (*P* < 0.001), subdural hematoma (*P* < 0.001), tSAH (*P* < 0.001), surgical treatment (*P* = 0.016), subdural effusion (*P* = 0.035), and ICU admission (*P* < 0.001) were significantly correlated with cognitive impairment after 3 months of mild-to-moderate TBI (Table 1). In addition, age (*P* = 0.003), GCS score (*P* < 0.001), >12 years of education (*P* = 0.047), diabetes mellitus (*P* = 0.005), hyperlipidemia (*P* = 0.004), injury side (bilateral sides vs. right side, *P* = 0.045), temporal lobe contusion (*P* = 0.001), subdural hematoma (*P* = 0.003), tSAH (*P* < 0.001), surgical treatment (*P* = 0.001), subdural effusion (*P* = 0.015), and ICU admission (*P* < 0.001) were significantly correlated with cognitive impairment after 12 months of mild-to-moderate TBI (Table 2).

**Table 1:** Univariable and multivariable logistic regression analyses on cognitive impairment after 3 months of mild-to-moderate TBI (*n* = 305).

	**TBI patients with cognitive impairment (*n =* 125)**	**TBI patients without cognitive impairment (*n =* 180)**	**Univariate analysis**		**Multivariate analysis**	
			**OR (95% CI)**	***P*** **-value**	**OR (95% CI)**	***P*** **-value**
Age (years)	57.10 ± 12.43	49.69 ± 15.97	1.036 (1.019, 1.054)	<0.001	1.036 (1.012, 1.059)	0.003^*^
Male sex (*n*, %)	93 (74.4%)	127 (70.6%)	1.213 (0.725, 2.028)	0.462		
GCS scores (points)	12 (10,14)	14 (12.25,15)	0.725 (0.647, 0.812)	<0.001	0.807 (0.681, 0.957)	0.014^*^
**Causes of injury (** ***n*** **, %)**						
Falling injury	53 (42.4%)	75 (41.7%)		0.401		
Traffic accident	66 (52.8%)	89 (49.4%)	1.049 (0.653, 1.686)	0.842		
Blunt injury	6 (4.8%)	16 (8.9%)	0.531 (0.195, 1.445)	0.215		
Years of education>12 (*n*, %)	13 (10.4%)	55 (30.6%)	0.264 (0.137, 0.508)	<0.001	0.223 (0.088, 0.566)	0.002^*^
Diabetes mellitus (*n*, %)	13 (10.4%)	10 (5.6%)	1.973 (0.836, 4.655)	0.121		
Hypertension (*n*, %)	32 (25.6%)	36 (20.0%)	1.376 (0.800, 2.369)	0.249		
Hyperlipemia (*n*, %)	39 (31.2%)	21 (11.7%)	3.434 (1.900, 6.205)	<0.001	3.249 (1.520, 6.941)	0.002^*^
**Injury side (** ***n*** **, %)**						
Right side	23 (18.4%)	61 (33.9%)		0.005		0.372
Left side	39 (31.2%)	57 (31.7%)	1.815 (0.967, 3.404)	0.063	1.738 (0.763, 3.957)	0.188
Bilateral sides	63 (50.4%)	62 (34.4%)	2.695 (1.488, 4.882)	0.001	1.628 (0.732, 3.619)	0.232
**Contusions (** ***n*** **, %)**						
Temporal lobe	82 (65.6%)	60 (33.3%)	3.814 (2.356, 6.175)	<0.001	2.606 (1.422, 4.776)	0.002^*^
Frontal lobe	67 (53.6%)	90 (50.0%)	1.155 (0.731, 1.825)	0.536		
Parietal lobe	7 (5.6%)	22 (12.2%)	0.426 (0.176, 1.031)	0.058	0.468 (0.165, 1.329)	0.154
Occipital lobe	10 (8.0%)	9 (5.0%)	1.652 (0.651, 4.192)	0.291		
**Hematoma (** ***n*** **, %)**						
Epidural hematoma	21 (16.8%)	47 (26.1%)	0.571 (0.322, 1.015)	0.056	0.820 (0.359, 1.872)	0.637
Subdural hematoma	69 (55.2%)	60 (33.3%)	2.464 (1.541, 3.940)	<0.001	1.226 (0.658, 2.284)	0.522
tSAH	92 (73.6%)	69 (38.3%)	4.485 (2.724, 7.383)	<0.001	2.837 (1.506, 5.346)	0.001^*^
Surgical treatment (*n*, %)	71 (56.8%)	77 (42.8%)	1.759 (1.109, 2.789)	0.016	1.736 (0.848, 3.553)	0.131
VER (*n*, %)	12 (9.6%)	31 (17.2%)	0.510 (0.251, 1.038)	0.063	0.166 (0.060, 0.458)	0.001^*^
Subdural effusion (*n*, %)	24 (19.2%)	19 (10.6%)	2.014 (1.050, 3.862)	0.035	1.198 (0.519, 2.768)	0.672
ICU admission (*n*, %)	68 (54.4%)	53 (29.4%)	2.859 (1.776, 4.602)	<0.001	2.285 (1.070, 4.881)	0.033^*^

TBI, traumatic brain injury; OR, odds ratio; GCS, Glasgow Coma Scale; tSAH, traumatic subarachnoid hemorrhage; VER, very early rehabilitation; ICU, intensive care unit. ^*^p < 0.05.

**Table 2:** Univariable and multivariable logistic regression analyses on cognitive impairment after 12 months of mild-to-moderate TBI (*n* = 305).

	**TBI patients with cognitive impairment (*n =* 52)**	**TBI patients without cognitive impairment (*n =* 253)**	**Univariate analysis**		**Multivariate analysis**	
			**OR (95% CI)**	***P*** **-value**	**OR (95% CI)**	***P*** **-value**
Age (years)	58.40 ± 10.74	51.56 ± 15.55	1.034 (1.011, 1.057)	0.003	1.035 (1.003, 1.068)	0.031^*^
Male sex (*n*, %)	41 (78.8%)	179 (70.8%)	1.541 (0.751, 3.161)	0.238		
GCS scores (points)	11 (10.13)	14 (12.15)	0.701 (0.609, 0.808)	<0.001	0.777 (0.637, 0.948)	0.013^*^
**Causes of injury (** ***n*** **, %)**						
Falling injury	21 (40.4%)	107 (42.3%)		0.848		
Traffic accident	28 (53.8%)	127 (50.2%)	1.123 (0.603, 2.091)	0.714		
Blunt injury	3 (5.8%)	19 (7.5%)	0.805 (0.218, 2.965)	0.744		
Years of education>12 (*n*, %)	6 (11.5%)	62 (24.5%)	0.402 (0.164, 0.986)	0.047	0.367 (0.117, 1.156)	0.087
Diabetes mellitus (*n*, %)	9 (17.3%)	14 (5.5%)	3.573 (1.455, 8.772)	0.005	4.443 (1.259, 15.681)	0.020^*^
Hypertension (*n*, %)	17 (32.7%)	51 (20.2%)	1.924 (0.999, 3.707)	0.051	0.972 (0.405, 2.331)	0.949
Hyperlipemia (*n*, %)	18 (34.6%)	42 (16.6%)	2.660 (1.374, 5.148)	0.004	2.224 (0.945, 5.230)	0.067
**Injury side (** ***n*** **, %)**						
Right side	9 (17.3%)	75 (29.6%)		0.129		0.824
Left side	16 (30.8%)	80 (31.6%)	1.667 (0.695, 3.999)	0.253	1.376 (0.473, 4.004)	0.558
Bilateral sides	27 (51.9%)	98 (38.7%)	2.296 (1.019, 5.172)	0.045	1.121 (0.418, 3.009)	0.820
**Contusions (** ***n*** **, %)**						
Temporal lobe	35 (67.3%)	107 (42.3%)	2.809 (1.495, 5.279)	0.001	1.637 (0.757, 3.540)	0.211
Frontal lobe	30 (57.7%)	127 (50.2%)	1.353 (0.740, 2.472)	0.326		
Parietal lobe	1 (1.9%)	28 (11.1%)	0.158 (0.021, 1.185)	0.073	0.172 (0.021, 1.437)	0.104
Occipital lobe	6 (11.5%)	13 (5.1%)	2.408 (0.871, 6.661)	0.090		
**Hematoma (** ***n*** **, %)**						
Epidural hematoma	9 (17.3%)	59 (23.3%)	0.688 (0.317, 1.494)	0.345		
Subdural hematoma	32 (61.5%)	97 (38.3%)	2.573 (1.393, 4.752)	0.003	1.222 (0.576, 2.590)	0.602
tSAH	41 (78.8%)	120 (47.4%)	4.131 (2.031, 8.401)	<0.001	2.449 (1.046, 5.735)	0.039^*^
Surgical treatment (*n*, %)	36 (69.2%)	112 (44.3%)	2.833 (1.495, 5.367)	0.001	2.473 (1.031, 5.932)	0.042^*^
VER (*n*, %)	7 (13.5%)	36 (14.2%)	0.938 (0.392, 2.240)	0.885		
Subdural effusion (*n*, %)	13 (25.0%)	30 (11.9%)	2.478 (1.189, 5.164)	0.015	1.700 (0.668, 4.327)	0.266
ICU admission (*n*, %)	34 (65.4%)	87 (34.4%)	3.604 (1.924, 6.750)	<0.001	1.891 (0.791, 4.521)	0.152

TBI, traumatic brain injury; OR, odds ratio; GCS, Glasgow Coma Scale; tSAH, traumatic subarachnoid hemorrhage; VER, very early rehabilitation; ICU, intensive care unit. ^*^p < 0.05.

We later introduced variables with a *p*-value of <0.10 identified using the univariable logistic regression analysis into the multivariable logistic regression analysis. Age (OR, 1.036; 95% CI [1.012,1.059]; *P* = 0.003), GCS scores (OR, 0.807; 95% CI [0.681, 0.957]; *P* = 0.014), >12 years of education (OR, 0.223; 95% CI [0.088, 0.566]; *P* = 0.002), hyperlipidemia (OR, 3.249; 95% CI [1.520, 6.941]; *P* = 0.002), temporal lobe contusions (OR, 2.606; 95% CI [1.422, 4.776]; *P* = 0.002), tSAH (OR, 2.837; 95% CI [1.506, 5.346]; *P* = 0.001), VER (OR, 0.166; 95% CI [0.060, 0.458]; *P* = 0.001), and ICU admission (OR, 2.285; 95% CI [1.070, 4.881]; *P* = 0.033) were independent risk factors for cognitive impairment after 3 months of mild-to-moderate TBI (Table 1). In addition, age (OR, 1.035; 95% CI [1.003, 1.068]; *P* = 0.031), GCS scores (OR, 0.777; 95% CI [0.637, 0.948]; *P* = 0.013), diabetes mellitus (OR, 4.443; 95% CI [1.259, 15.681]; *P* = 0.020), tSAH (OR, 2.449; 95% CI [1.046, 5.735]; *P* = 0.039), and surgical treatment (OR, 2.473; 95% CI [1.031, 5.932]; *P* = 0.042) were independent risk factors for cognitive impairment after 12 months of mild-to-moderate TBI (Table 2).

### Two nomograms to predict cognitive impairment after 3 and 12 months of mild-to-moderate TBI

**Figure 1:**
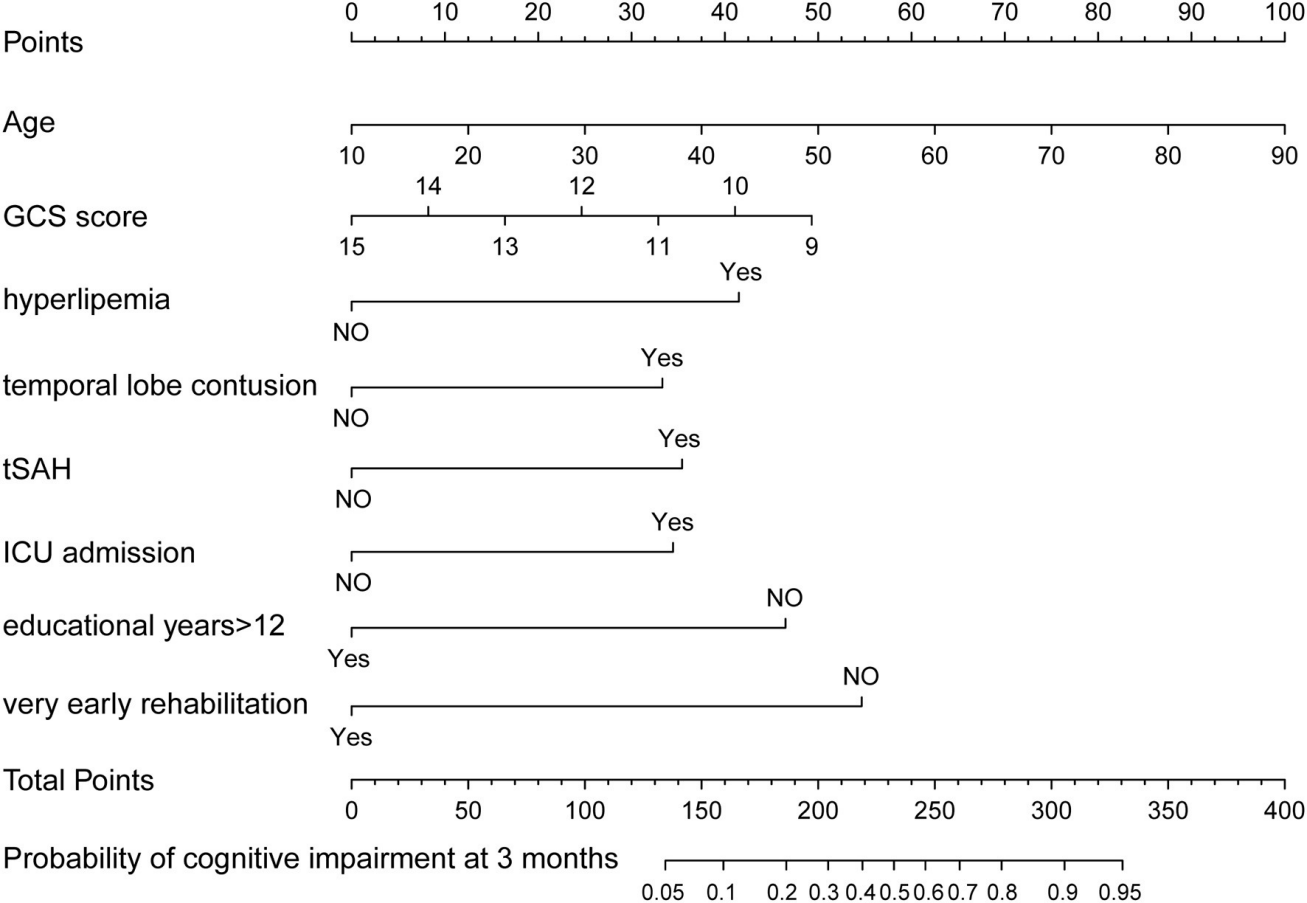
Nomogram predicting cognitive impairment at 3 months after mild-to-moderate TBI.

**Figure 2:**
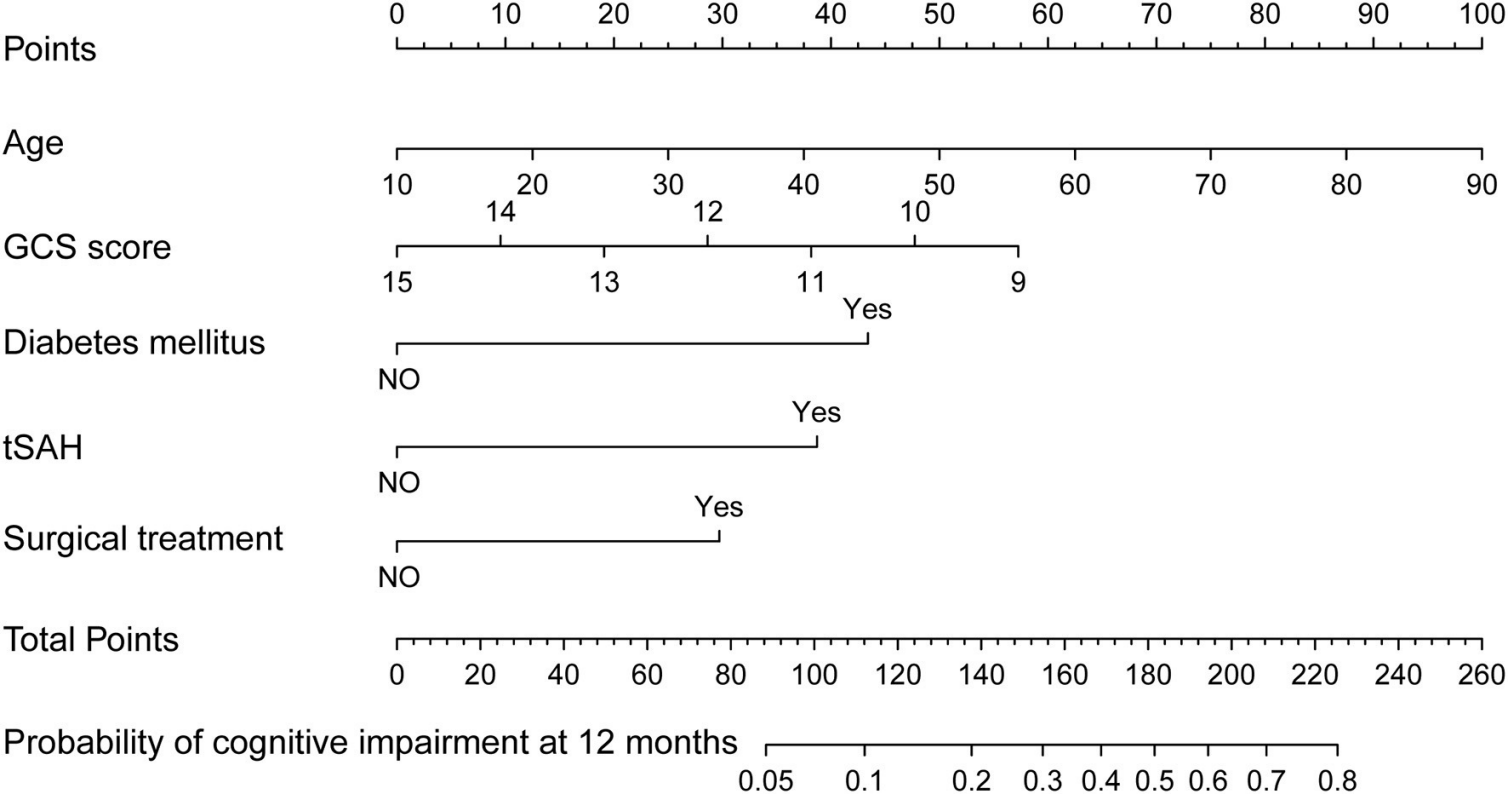
Nomogram predicting cognitive impairment at 12 months after mild-to-moderate TBI.

**Figure 3:**
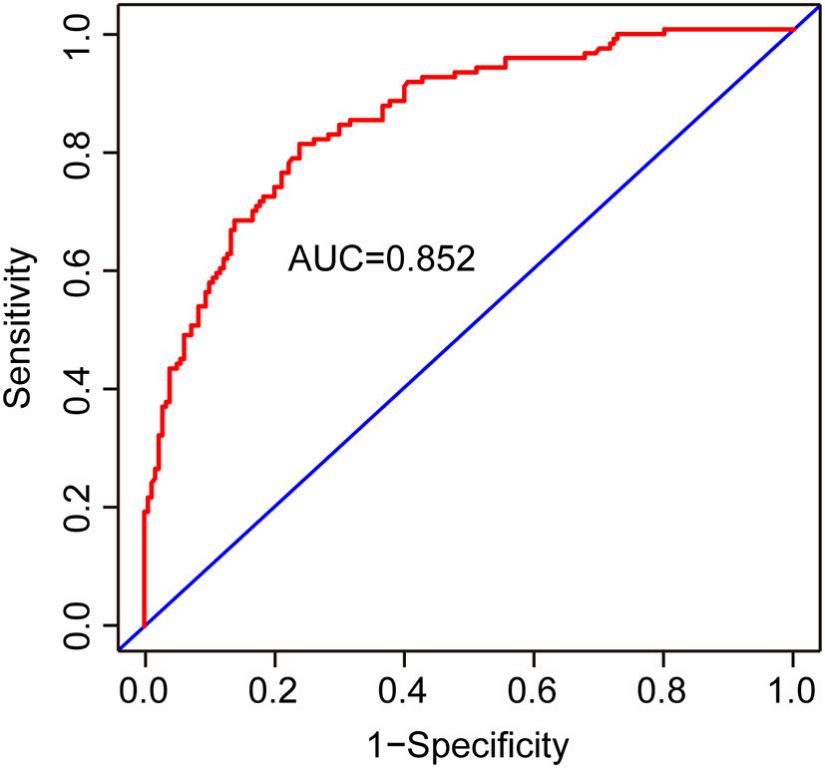
ROC curves to validate the discrimination of the nomogram predicting cognitive impairment 3 months after mild-to-moderate TBI.

**Figure 4:**
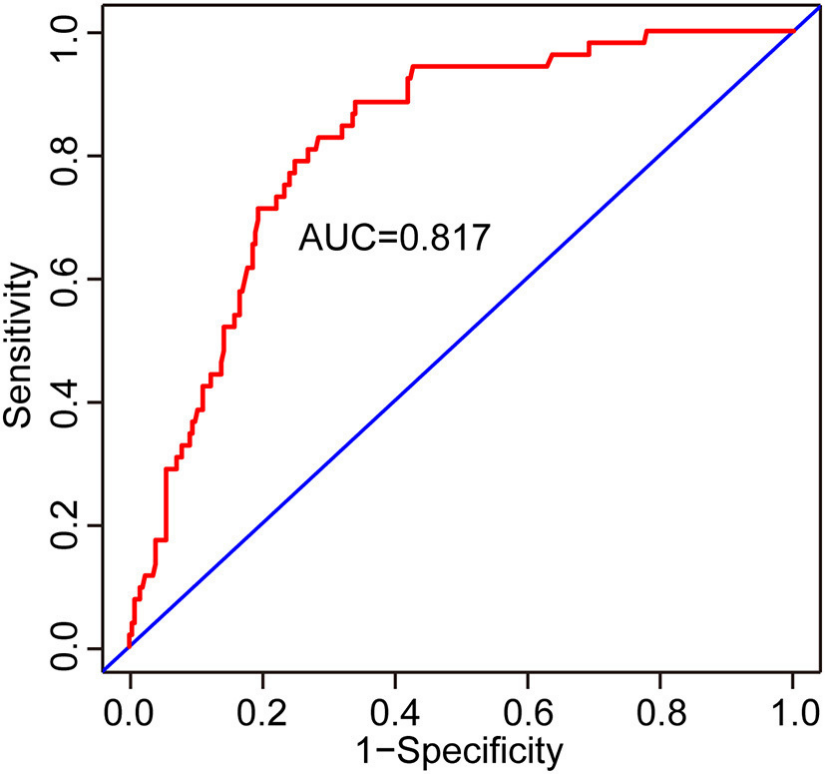
ROC curves to validate the discrimination of the nomogram predicting cognitive impairment at 12 months after mild-to-moderate TBI.

**Figure 5:**
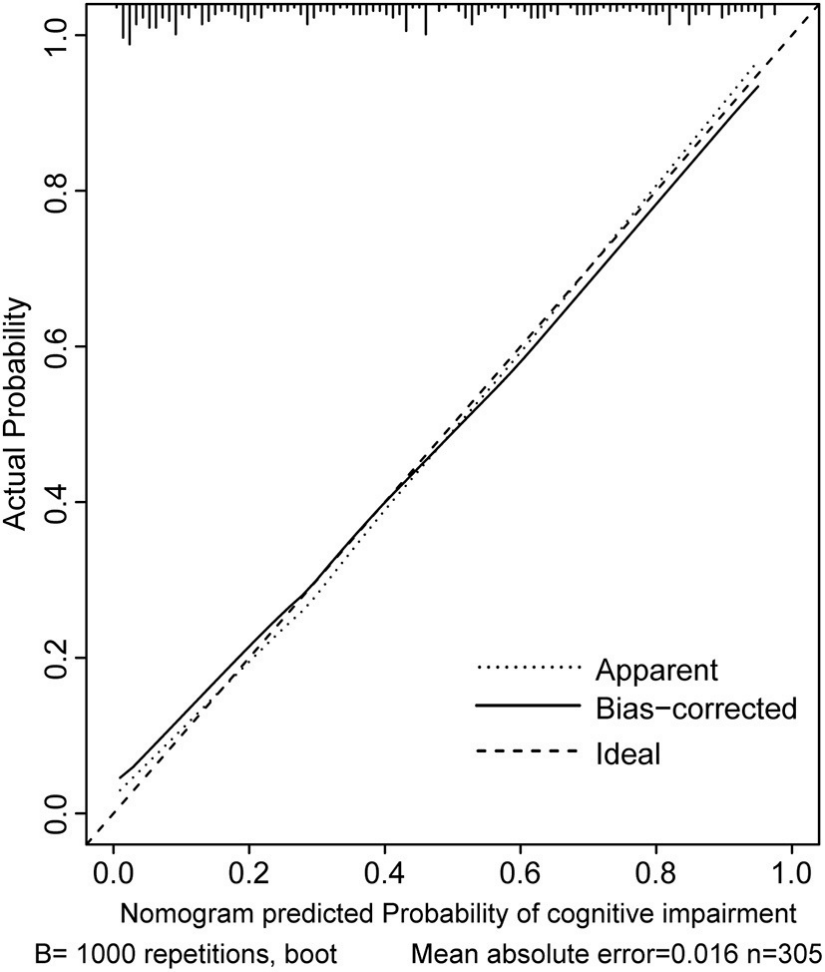
Calibration curves of the nomogram for predicting cognitive impairment at 3 months after mild-to-moderate TBI.

**Figure 6:**
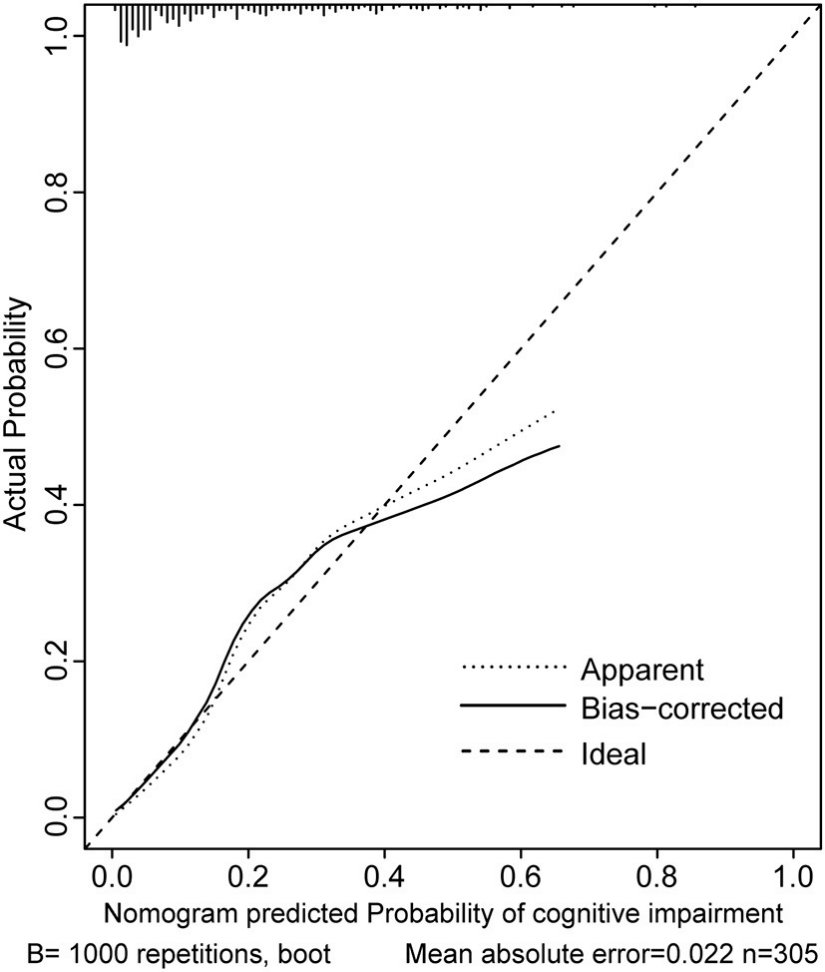
Calibration curves of the nomogram for predicting cognitive impairment at 12 months after mild-to-moderate TBI.

Two nomograms were then created based on risk factors identified using the multivariable logistic regression analysis to predict cognitive impairment after 3 and 12 months of mild-to-moderate TBI (Figures 1, 2). The total score was the sum of the points for each covariate in the nomogram and corresponded to the predicted probability of the outcome of interest. The AUCs of the nomogram to predict cognitive impairment after 3 and 12 months of mild-to-moderate TBI were 0.852 (95% CI [0.810, 0.895]) and 0.817 (95% CI [0.762, 0.873]), respectively, suggesting a good discriminative ability of the two nomograms (Figures 3, 4). The C-statistics of a nomogram to predict cognitive impairment after 3 months of mild-to-moderate TBI was 0.834 by internal validation using bootstrapping with 1,000 iterations. The C-statistics of a nomogram to predict cognitive impairment after 12 months of mild-to-moderate TBI was 0.799 after bootstrapping. The actual and predicted probabilities of cognitive impairment on the Y-axis and X-axis were plotted, respectively. The calibration curves showed an acceptable goodness-of-fit of the nomograms (Figures 5, 6).

## Discussion

Cognitive impairment is a common complication following mild-to-moderate TBI, which may last for a long term. de Boussard et al. (2005) have reported that 26% of mild TBI patients develop cognitive impairment 3 months later. Consistently, Skandsen et al. (2010) have illustrated that 43% of moderate TBI patients have cognitive impairment at 3 months post-TBI. An observational study in Malaysia has shown that 19.2% of mild TBI patients and 39.3% of moderate TBI patients presented cognitive impairment after 3 months of injury (Othman et al., 2022). In the present study, the incidences of cognitive impairment after 3 months of mild and moderate TBI were 28.9% and 60.9%, respectively. Both higher incidences, compared to those previously reported, may be attributed to the differences in age, cause of injury, and injury site. Few previous studies have focused on cognitive impairment 1 year after mild-to-moderate TBI. Schneider et al. (2022) have reported that 10.1% of mild TBI patients have cognitive impairment even after 1 year of injury. Similarly, our data revealed that the incidence of cognitive impairment after 12 months of mild-to-moderate TBI was 17.0%, i.e., 8.4% in mild and 31.3% in moderate TBI patients.

We later created two nomograms to predict cognitive impairment in mild-to-moderate TBI patients after 3 and 12 months of TBI, based on independent risk factors identified using the multivariable logistic regression analysis. Through a retrospective analysis of the clinical data of 305 mild-to-moderate TBI patients, we found that older age, low GCS score, hyperlipidemia, temporal lobe contusion, tSAH, and ICU admission were independent risk factors for cognitive impairment after 3 months of mild-to-moderate TBI, while >12 years of education and VER were protective factors. In addition, older age, low GCS score, diabetes mellitus, tSAH, and surgical treatment were independent risk factors for cognitive impairment after 12 months of mild-to-moderate TBI.

Age, GCS score, and tSAH were all closely linked with cognitive impairment at 3 and 12 months after mild-to-moderate TBI. Leukoaraiosis and poor neuroplasticity in elderly patients can increase the risk of cognitive impairment after TBI (Nguyen et al., 2019). Both aging and TBI cause the loss of brain volume and a decline of white matter integrity, and their additive effect, notably, prolongs the negative influence of cognitive impairment on elderly patients with TBI (Farbota et al., 2012; Arenth et al., 2014; Kim et al., 2021). The hippocampus is a region responsible for cognitive function, especially memory function. Biological functions of the hippocampus can be largely impaired by subarachnoid hemorrhage, the subsequent middle cerebral artery spasm can reduce blood supply, block synaptic neurotransmission, and damage plasticity (Tariq et al., 2010; Regnier-Golanov et al., 2022). Neuroinflammation and oxidative stress in hippocampal neurons secondary to subarachnoid hemorrhage also contribute to cognitive impairment (Han et al., 2014; Hu et al., 2021). In patients with aneurysmal subarachnoid hemorrhage, abnormal changes in the microstructure of the white matter result in cognitive impairment 3 months after the onset (Reijmer et al., 2018).

Cognitive outcomes vary a lot after contusion and hemorrhage in different brain regions. Martin et al. (2017) have demonstrated that the volume of frontal lobe contusion is not linked with cognitive outcomes, while a larger volume of temporal lobe contusion predicts a worse cognitive function after 6 months of injury. A close correlation is identified between the hemorrhage site and the incidence of dementia within 6 months of cerebral hemorrhage rather than delayed dementia after 6 months (Biffi et al., 2016). Our results showed that mild-to-moderate TBI patients with temporal lobe contusions had a higher risk of cognitive impairment at 3 months post-TBI, but cognitive impairment at 12 months was not significantly correlated with the site of contusion.

Hypertension, diabetes mellitus, and hyperlipidemia have not been the focus of research concerning cognitive impairment following TBI. Our data demonstrated that hyperlipidemia and diabetes mellitus were independent risk factors of cognitive impairment 3 and 12 months after mild-to-moderate TBI, respectively. The risk of cognitive impairment in patients with diabetes mellitus is 1.5–2 times higher than that in patients without diabetes mellitus (Cukierman et al., 2005). Lachmann et al. (2018) have found that diabetes mellitus is linked with an increased risk of postoperative cognitive impairment rather than hypertension. The negative influence of diabetes mellitus on cognitive function can be attributed to hippocampal atrophy and cerebral microvascular damage (van Elderen et al., 2010; Hayashi et al., 2011; Vuletic et al., 2013). Hyperlipidemia increases the incidence of carotid intimal thickening, and meanwhile, TBI may accelerate the process of atherosclerosis (Wang et al., 2018). A synergic effect of hyperlipidemia and TBI causes carotid atherosclerosis, and the subsequent cerebrovascular insufficiency or cerebral microinfarct poses a long-term impact on cognitive function.

Mild-to-moderate TBI patients who were admitted to the ICU were more likely to have cognitive impairment 3 months after TBI than those without an ICU admission, which may be linked with ICU-acquired delirium. A much higher incidence of delirium is detected in patients admitted to the ICU, which in TBI patients, can be as high as 60% (Wilson et al., 2023). Delirium is one of the important causes of long-term cognitive decline (Goldberg et al., 2020).

We further found that >12 years of education and VER were identified as protective factors for cognitive impairment at 3 months after mild-to-moderate TBI, which, however, did not influence cognitive function at 12 months. A high level of education provides a strong cognitive reserve to cope with TBI-induced physical and psychological challenges (Almeida-Meza et al., 2022). Cognitive impairment following TBI is found to be associated with the level of apolipoprotein E ε4 (ApoE-ε4), which reduces brain metabolism in the medial temporal and prefrontal lobe of TBI patients (Hellstrøm et al., 2022). Interestingly, a high level of education is able to reverse the negative influence of ApoE-ε4 on brain metabolism (Arenaza-Urquijo et al., 2015). VER is an emerging concept of rehabilitation. Currently, clinical data supporting the role of VER in TBI are controversial. In a retrospective cohort study involving acute stroke patients in Japan, VER is validated to reduce the disability rate of stroke (Matsui et al., 2010). However, a multi-center randomized controlled trial (RCT) illustrates that VER does not significantly improve the quality of life and communication skills of stroke patients compared with conventional nursing care (Cumming et al., 2019; Godecke et al., 2021). In the present study, VER protected cognitive function in mild-to-moderate TBI patients possibly due to selection biases resulting from differences in comorbidities of other injuries, duration of disease stabilization, and the willingness to cooperate with rehabilitation exercises. Rigorous-designed RCTs are needed in future to analyze the clinical benefits of VER for mild-to-moderate TBI patients.

We finally created two nomograms to predict risk factors for cognitive impairment after 3 and 12 months of mild-to-moderate TBI, in which conventional demographic, clinical, and radiological data were incorporated. Both were accurate to predict cognitive impairment after TBI, thus making it possible to design an individualized and timely therapeutic strategy to prevent cognitive dysfunction. There are several limitations in the present study. First of all, we excluded TBI patients with severe combined injuries, which limited the application of the prediction models. Second, we did not retrospectively analyze electroencephalogram (EEG) data and laboratory testing data in TBI patients, which may influence the discriminative ability of the nomograms. Third, it was a single-center retrospective study that lacked external validation. Our findings should be further validated in multi-center clinical studies.

## Conclusion

Age, GCS score, >12 years of education, hyperlipidemia, temporal lobe contusion, tSAH, VER, and ICU admission are independent risk factors for cognitive impairment after 3 months of TBI; meanwhile, age, the GCS scores, diabetes mellitus, tSAH, and surgical treatment are independent risk factors of cognitive impairment after 12 months of TBI. Two nomograms, based on both groups of factors, respectively, showed strong discriminative abilities for cognitive impairment, which may be used to assist clinical management of cognitive impairment following TBI.

## Data availability statement

The original contributions presented in the study are included in the article/supplementary material, further inquiries can be directed to the corresponding authors.

## Ethics statement

The studies involving human participants were reviewed and approved by Ethics Committee of the First Affiliated Hospital of Nanjing Medical University. The patients/participants provided their written informed consent to participate in this study.

## Author contributions

XiW, XH, and XiaW conceived and designed the study. XiW, XG, XL, and YS collected the dates. BH and XH analyzed the results and wrote the manuscript. YS and LL reviewed and edited the manuscript. All authors contributed to the article and approved the submitted version.
